# Self-Reported Mental Health and Lifestyle Behaviour During the COVID-19 Pandemic in the Czech Population: Evidence From Two Cross-Sectional Surveys

**DOI:** 10.3389/ijph.2022.1604395

**Published:** 2022-05-12

**Authors:** Andrea Dalecká, Hana Tomášková, Hana Šlachtová, Dagmar Skýbová, Ratislav Mad’ar

**Affiliations:** ^1^ Department of Epidemiology and Public Health, Faculty of Medicine, University of Ostrava, Ostrava, Czechia; ^2^ Centre for Epidemiological Research, Faculty of Medicine, University of Ostrava, Ostrava, Czechia; ^3^ Institute of Public Health in Ostrava, Ostrava, Czechia

**Keywords:** mental health, COVID-19, physical activity, health behaviours, quality of sleeping, dietary habits

## Abstract

**Objectives:** Evidence of the impact of COVID-19 pandemic on mental and physical health behaviours is limited. This study presents results of two cross-sectional surveys on mental health changes and its consequences on healthy and unhealthy lifestyle behaviours.

**Methods:** An online survey was distributed during Spring 2020 (*N* = 9,168) and Autumn 2020 (*N* = 1,042) in the Czech Republic. Differences in mental health observed in both surveys were evaluated using Mann-Whitney test and logistic regressions were used to examine demographic and socio-economic determinants of COVID-19-related mental health issues and resulting healthy and unhealthy lifestyle behaviours.

**Results:** In multivariable models, the youngest individuals, females, people with increased work demands and participants with a reduced personal income due to the COVID-19 pandemic were all negatively associated with self-reported mental health issues (*p* < 0.05). A worsened quality of sleep, dietary habits, physical activity and unhealthy behaviours were highly associated with affected mental health in the models adjusted for potential covariates (*p* < 0.05).

**Conclusion:** Taken together, these findings suggest that health promotion strategies directed to individuals who are at risk should be encouraged to adopt and/or maintain positive health-related behaviours.

## Introduction

The ongoing novel coronavirus disease (COVID-19) pandemic has become an unprecedented public health and economic threat worldwide that affects all individual of all ages and all countries [1, 2]. Due to the unrestrained spread of the disease and limited vaccine availability, many countries have been adopted strict restrictions that are designed to protect their citizens [3–6].

Besides personal protective practices (hand washing, wearing face masks, keeping distance), there has also been a number of measures that influence the daily life of a population such as a stay-at-home rule, social distancing, the closure of educational institutions and limitations of leisure-time activities [7]. Although such interventions are necessary to mitigate the transmission of the infection, this recommended isolation is likely to have a harmful effect on mental health and well-being as it might produce psychological distress, fear and loneliness [8, 9]. Moreover, it should be emphasised that the pandemic has also indirectly affected the economic and social stability of individuals and communities [10, 11].

Evaluating an individual’s mental well-being and its resulting behavioural lifestyle response is, therefore, essential for developing the effective management of the global pandemic [12, 13]. Socio-demographic predictors, occupational and economical disbalance and personal beliefs are factors that can affect mental health [14, 15]. For instance, women, young people, people living alone and front-line workers evaluated their mental health issues (anxiety, depression) as being significantly worse in several studies [14, 16, 17]. The preliminary results of this study showed that subjectively perceived mental health was not evaluated as being momentous. Nevertheless, the first observation was conducted at the beginning of the COVID-19 outbreak in the Czech Republic (Spring 2020), and therefore the effect of the lockdown and restrictions had not affected mental health for a sufficient length of time. Mental health was significantly affected by age, gender, marital status, increased work demands and socio-economic factors according to the preliminary results [18].

Most of the existing research on the pandemic-related consequences has focused on the immediate (one time-point) situation and does not reflect the possible development of risk perception and related psychological implications over time. The prolonged pandemic has inevitably brought financial uncertainties and long-lasting isolation from family and friends that have escalated mental health burdens. In the light of this situation, we present the results of a comparative analysis of public mental health from the first survey conducted in April 2020 (hereinafter “Spring 2020”) and the second survey conducted in November-December 2020 (hereinafter “Autumn 2020”) in the Czech population in order to strengthen the research evidence that is related to the individual determinants of mental health and its consequences.

Emerging evidence has suggested that the mental health instability that has been caused by social isolation and stress during the COVID-19 pandemic [19, 20] might consequently influence the negative implications of the pandemic, e.g., a decrease in the compliance with the mitigating measures [21] and an increase in the unhealthy dietary and lifestyle factors [22, 23]. The effect might become even stronger if the trust and effective communication from the government and public health policy representatives are not sufficient and effective [12, 15, 24].

Recent studies have reported increases in the addictive lifestyle behaviours such as smoking and alcohol use [25–27]. By contrast, around a quarter of the United States adult participants has reduced their use of cigarettes and e-cigarettes [27]. Moreover, both negative and positive effects of the COVID-19 lockdown have been reported in relation to dietary habits. The number of snacks being consumed and the frequency of eating has increased, especially in young people [28, 29], while favourable dietary changes including an increase in the consumption fresh products and home cooking have also been reported [30–32]. With respect to the limited opportunities for doing physical activities due to the closure of sport facilities, it has been suggested that there has been an increase in sedentary behaviours and a decrease in daily physical activities. Meanwhile, very few studies have reported an increase in physical activities among healthy adults [33, 34]. Diverse results have also been observed in the quality of sleep during the pandemic [25, 34–36].

It should be pointed out that stress and a worsened state of mental well-being are considered to be critical risk factors for negative lifestyle changes [25, 37]. Moreover, the observed lifestyle changes during the pandemic that have emerged have affected the various population groups in different ways. Therefore, in this population-based study, we examined the demographic, socio-economic and behavioural determinants of self-reported changes in healthy (i.e., diet, physical activity, quality of sleeping) and unhealthy (i.e., smoking, alcohol use, screen time) lifestyle behaviours during the periods of COVID-19 lockdowns. By identifying the individual-level determinants and at-risk groups, lifestyle and mental health interventions during pandemics might be targeted effectively.

## Methods

Two cross-sectional questionnaire surveys were conducted during the first (Spring 2020) and the second (Autumn 2020) wave of the spread of the coronavirus disease in the Czech Republic. The results presented here were collected during a 4-week period in both survey campaigns. The web questionnaire survey was announced on national TV and radio stations that have nationwide coverage. Therefore, individuals from all of the regions of the Czech Republic could participate in the survey. Respondents were also recruited through the website of the University of Ostrava where the link to the online questionnaire was available during the entire duration of the emergency. All procedures performed in the survey were in accordance with the ethical standards of the Ethics Committee of the Faculty of Medicine, University of Ostrava (Reference number: 30/2020). The gathered data were analysed and processed according the General Data Protection Regulation. This study was voluntary and strictly anonymous, therefore, as no identifying details (names, dates of birth, identity numbers, addresses etc.) were collected from the participants and even no personal contact with the respondents was performed, no informed consent of individuals was required for this kind of study.

### Mental Health Changes

Subjectively perceived mental health was observed in both surveys and it has been hypothesized that it might develop over time of pandemic. In this cross-sectional study, differences between the first (Spring 2020) and the second (Autumn 2020) surveys were assessed for mental health by using Mann-Whitney test (on 5% significance level). Mental health was evaluated subjectively by answering the question “How would you evaluate your mental health in relation to COVID-19?”. The variable was evaluated as average value of the 5-point (1 = “very good; …; 5 = “very bad”) Likert scale. The binary variable “affected mental health” was created by grouping the categories “bad” and “very bad” (coded as 1) and categories “very good”, “good” and “average” (coded as 0). No specific mental outcomes (e.g., anxiety, depression or loneliness) were separately evaluated. In case the respondent evaluated his/her mental health as “bad” or “very bad”, the question “What is the reason of your worsened mental health?” was open for answer. The question was predefined with 13 reasons (Supplemental Material 1).

The crude (univariate) and multivariable ordinal logistic regression on the significance level of 5% were conducted to analyse whether the individual demographic and socio-economic factors (gender, age, education, marital status, increased work demands, decreased personal income and living with a child <aged 15) were associated with mental health in both observations (see Supplemental Material 2 for individual and socio-economic variables characteristics). First, the association of each variable on outcome was tested separately (crude model). Next, all the variables were considered as covariates and examined in separate multivariable regression models.

### Healthy and Unhealthy Lifestyle Behavioural Changes

The second survey was supplemented with extra questions that were related to healthy (quality of sleeping, dietary habits and physical activity) and unhealthy (smoking, alcohol use, watching TV and playing digital games) lifestyle changes that had developed during the pandemic. The lifestyle variables were not collected in the first survey as no substantial changes were expected due to the very short period after the outbreak of the pandemic.

The self-reported changes in healthy lifestyle behaviour were indicated by asking for the quality of the variables (“better”, “worse” or “stayed the same”), and whether changes in unhealthy lifestyle behaviour were indicated by asking for a frequency (“more often”, “less often” or “stayed the same”) of the variables.

The quality of sleeping, dietary habits and physical activity were analysed separately, whereas the unhealthy addictive behaviour (smoking and alcohol use) as well as sedentary behaviour (time spent watching TV and time spent playing digital games) were analysed as a joint variable. Thus, a worsening in any of the observed unhealthy or addictive variables was considered to be a change to an addictive, respectively sedentary behaviour. These two unhealthy behavioural changes variables were, therefore, recoded by creating a new merged variables called “addictive behaviour” and “sedentary behaviour”.

Crude (univariate) logistic regression analyses were conducted to examine the associations between each of the demographic and socio-economic variables (as listed above) as well as the impact of affected mental health that was observed in the second survey on the healthy and unhealthy behavioural lifestyle changes that had developed during the pandemic. The reference category for the dependent variables was “no change/positive change”. All respondents who reported “not relevant” in lifestyle variables were not included into analysis. Next, all of these variables were considered as covariates and examined in separate multivariable regression models as they are likely to have a confounding effect based on a recent research.

The results are expressed as the crude and adjusted odds ratios (OR) with 95% confidence intervals (CI). The data was analysed using the SW STATA version 15.

## Results

### Sample Characteristics

Data were obtained from 9,168 respondents during the first survey, which was performed in Spring 2020 and from 1,042 respondents during the second survey, which was performed in November/December 2020. As is shown in Table 1, the study sample for both surveys were predominantly female (76.8% in Spring 2020 vs. 66.5% in Autumn 2020), middle-aged individuals (64.0% vs. 66.6%) and respondents with a university education (54.4% vs. 56.4%). Most of the respondents were married or were living in a partnership (64.2% vs. 66.9%).

**TABLE 1:** Sociodemographic, occupational and mental health characteristics. Czech Republic, Spring 2020 and Autumn 2020.

Sociodemographic characteristics	Spring 2020		Autumn 2020	
	*n*	%	*n*	%
Total	**9,168**		**1,042**	
Gender				
Male	2,108	23.2	349	33.5
Female	6,966	76.8	693	66.5
Age				
15–29 years	2,281	25.2	161	15.5
30–44 years	3,629	40.1	375	36.0
45–59 years	2,156	23.9	319	30.6
60 or more years	973	10.8	186	17.9
Education				
Primary/lower second	938	10.3	98	9.4
Upper secondary	3,208	35.3	356	34.2
University/Tertiary	4,939	54.4	588	56.4
Marital status				
Married/partnership	5,889	64.2	697	66.9
Single/divorced/widowed	3,279	35.8	345	33.1
Size of municipality				
Village/small town (<2K inhabitants)	1,469	16.2	211	20.2
Small town (2K–10K inhabitants)	1,382	15.2	155	14.9
Middle town (10K–100K inhabitants)	2081	22.9	300	28.8
City (>100K inhabitants)	3,048	33.5	254	24.4
Capital city (Prague >1,000K inhabitants)	1,108	12.2	122	11.7
Increased work demands				
Yes	1764	19.2	238	22.8
Decreased personal income				
Yes	1,625	17.7	149	14.3
Living with someone < age 15				
Yes	3,116	34.5	399	40.7

The bold values on the top of the table represent total size of the samples in Spring and Autumn observations.

### Mental Health Changes During the COVID-19 Pandemic

The mean values of the subjectively evaluated mental health increased from 2.48 (median: 2; IQR: 2–3) to 2.67 (median: 3; IQR: 2–4) on the five-point Likert scale between Spring and Autumn 2020. We found a significant difference between the time points in the observed variable (*p*-value < 0.001). The respondents evaluated their mental health during COVID-19 pandemic as “bad” or “very bad” (four and five points on the Likert scale, respectively) – 16.4% in the first wave and 26.6% in the second wave of the pandemic. The main reasons for the worsened mental health were associated with the lack of meaningful activities (19.7%), isolation from relatives and friends (18.6%) and concerns about an uncertain future (17.2%). In contrast, the self-reported risk perception substantially decreased between Spring and Autumn (*p*-value < 0.001).

**FIGURE 1:**
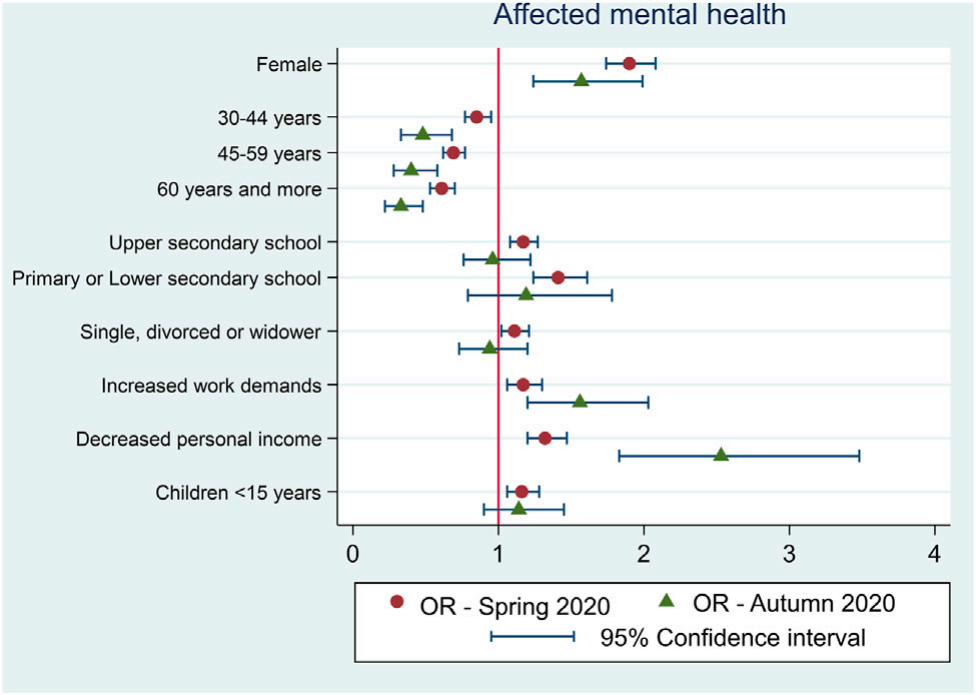
Multivariable (adjusted) regression models of the associations between demographic and socio-economic determinants and mental health. Czech Republic, Spring 2020 and Autumn 2020. Note: The reference categories were men, age 15-29 years, university/tertiary education, married/partnership.

As Figure 1 and Table 2 show, the full adjusted analysis revealed that the youngest individuals (age 15–29), females, individuals with increased work demands and those with decreased personal income due to the COVID-19 pandemic were all negatively associated with a self-reported worsened mental health in the first and in the following observation. The association between decreased personal income and affected mental health significantly strengthen in Autumn compared to Spring observation.

**TABLE 2:** Associations between demographic and socio-economic determinants and mental health. Czech Republic, Spring 2020 and Autumn 2020.

	Mental health (Spring 2020)		Mental health (Autumn 2020)	
Factors	Crude OR (95% CI)	Adjusted OR (95% CI)	Crude OR (95% CI)	Adjusted OR (95% CI)
Gender (Ref. cat. - Men)				
Female	1.91 ** (1.75–2.09)	1.90 ** (1.74–2.08)	1.54 ** (1.22–1.94)	1.57 ** (1.24–1.99)
Age (Ref. cat. – 15–29 years)				
30–44 years	0.88 * (0.8–0.97)	0.85 * (0.77–0.95)	0.60 * (0.43–0.83)	0.48 ** (0.33–0.68)
45–59 years	0.72 ** (0.65–0.80)	0.69 ** (0.62–0.77)	0.51 ** (0.36–0.71)	0.40 ** (0.28–0.58)
60 years or more	0.56 ** (0.49–0.64)	0.61 ** (0.53–0.70)	0.33 ** (0.22–0.48)	0.33 ** (0.22–0.48)
Education (Ref. cat. – University/Tertiary)				
Upper secondary school	1.17 ** (1.08–1.27)	1.17 ** (1.08–1.27)	0.95 (0.75–1.2)	0.96 (0.76–1.22)
Primary/Lower secondary school	1.29 ** (1.14–1.47)	1.41 ** (1.24–1.61)	1.15 (0.78–1.71)	1.19 (0.79–1.78)
Marital status (Ref. cat. – Married/Partnership)				
Single, divorced or widower	1.08 * (1.00–1.17)	1.11 * (1.02–1.21)	0.70 * (0.52–0.96)	0.94 (0.73–1.2)
Increased work demands				
Yes	1.06 (0.97–1.16)	1.17 * (1.06–1.30)	1.44 * (1.12–1.87)	1.56 * (1.20–2.03)
Decreased personal income				
Yes	1.38 ** (1.25–1.52)	1.32 ** (1.20–1.47)	2.30 ** (1.69–3.13)	2.53 ** (1.83–3.48)
Living with someone < age 15				
Yes	1.21 ** (1.12–1.31)	1.16 * (1.06–1.28)	1.16 (0.94–1.44)	1.14 (0.90–1.45)

Note: Univariate and multivariable (adjusted) regression models showing the association of demographic and socio-economic determinants and mental health during the observations (Spring and Autumn 2020). *p < 0.05, **p < 0.001.

Compared to the first observation, which was conducted at the beginning of the lockdown, the significant negative association of the university education, being single/divorced/widowed and taking care of child aged <15 were no longer significant.

### Healthy and Unhealthy Lifestyle Behavioural Changes

Considering healthy lifestyle behaviours, 30.2, 20.7 and 65.7% of the respondents reported a worsened quality of sleeping, dietary habits and physical activity, respectively (Table 3). An improvement in healthy lifestyle behaviours was very rare, only 5.1, 8.9 and 5.0% respondents, respectively, reported a better quality of sleeping, dietary habits and physical activity. On the other hand, a worsening of at least one of the unhealthy lifestyle factors was observed in 43.7% of the respondents. A substantial negative change in unhealthy behaviours was especially associated with the time spent watching TV/movies (39.9%) or playing digital games (34.9%) in the participants who reported that these activities were relevant. Moreover, the frequency of smoking increased in 24.1% of the smokers. By contrast, both a positive and a negative incidence of alcohol use was observed (22.3% compared to 19.4%, respectively) (Table 3).

**TABLE 3:** Healthy and unhealthy behavioural lifestyle characteristics. Czech Republic, Autumn 2020.

Lifestyle variables (*n* = 1,042)	*n*	%
Quality of sleeping		
1 = better	53	5.1
2 = no change	672	64.7
3 = worse	314	30.2
Dietary habits		
1 = better	92	8.9
2 = no change	731	70.4
3 = worse	215	20.7
Physical activity		
1 = better	51	5.0
2 = no change	299	29.1
3 = worse	679	66.0
Smoking		
0 = not relevant	801	77.2
1 = less frequent	29	2.8
2 = no change	151	14.5
3 = more often	57	5.5
Alcohol use		
0 = not relevant	407	39.3
1 = less frequent	140	13.5
2 = no change	367	35.4
3 = more often	122	11.8
Screen time (PlayStation, PC games etc.)		
0 = not relevant	699	67.8
1 = less frequent	26	2.5
2 = no change	190	18.4
3 = more often	116	11.3
Screen time (TV, movies etc.)		
0 = not relevant	112	10.9
1 = less frequent	54	5.2
2 = no change	498	48.3
3 = more often	367	35.6

#### Quality of Sleeping

As Table 4 shows, females, individuals with increased work demands and those with decreased personal income due to the COVID-19 pandemic respondents with worsened mental health were all more likely to have a lower quality of sleeping. All of these factors were significant in both the univariate and multivariable models. The most visible association was observed in respondents whose mental health had deteriorated as a consequence of the COVID-19-related restrictions and changes in their lives (OR = 4.82; CI: 3.53–6.58).

**TABLE 4:** Associations between demographic, socio-economic and affected mental health determinants and healthy and unhealthy behavioural lifestyle. Czech Republic, Autumn 2020.

	Sleeping quality		Dietary habits		Physical activity		Addictive behaviour^a^		Sedentary behaviour^b^	
Factors	Crude OR (95%CI)	Adjusted OR^c^(95% CI)	Crude OR (95%CI)	Adjusted OR^c^ (95% CI)	Crude OR (95%CI)	Adjusted OR^c^ (95% CI)	Crude OR (95%CI)	Adjusted OR^c^ (95% CI)	Crude OR (95%CI)	Adjusted OR^c^ (95% CI)
Gender (Ref. cat. - Men)										
Women	1.74 ** (1.30–2.35)	1.67 ** (1.21–2.30)	0.95 (0.69–1.30)	0.85 (0.61–1.19)	1.06 (0.81–1.39)	0.98 (0.74–1.30)	0.81 (0.57–1.16)	0.79 (0.54–1.15)	1.08 (0.83–1.41)	1.19 (0.90–1.58)
Age (Ref. cat. – 15–29 years)										
30–44 years	1.27 (0.84–1.91)	1.41 (0.87–2.28)	0.73 (0.48–1.11)	0.72 (0.44–1.16)	0.80 (0.53–1.20)	0.74 (0.48–1.16)	0.77 (0.49–1.21)	0.90 (0.54–1.50)	0.38 ** (0.26–0.55)	0.50 * (0.33–0.75)
45–59 years	1.21 (0.79–1.84)	1.41 (0.87–2.28)	0.63 * (0.41–0.99)	0.68 (0.42–1.11)	0.69 (0.46–1.04)	0.69 (0.44–1.07)	0.36 ** (0.21–0.61)	0.42 * (0.24–0.74)	0.33 ** (0.22–0.49)	0.38 ** (0.25–0.58)
60 and more years	0.92 (0.57–1.49)	1.58 (0.93–2.69)	0.38 * (0.22–0.66)	0.51 * (0.28–0.90)	0.60 * (0.38–0.95)	0.71 (0.44–1.13)	0.21 ** (0.10–0.43)	0.28 * (0.14–0.59)	0.32 ** (0.21–0.50)	0.38 ** (0.24–0.61)
Education (Ref. cat. – University/Tertiary)										
Upper secondary	0.86 (0.64–1.15)	0.84 (0.61–1.15)	1.05 (0.77–1.45)	1.06 (0.76–1.49)	0.79 (0.60–1.04)	0.78 (0.59–1.04)	0.89 (0.61–1.30)	0.89 (0.59–1.33)	1.10 (0.84–1.45)	1.07 (0.80–1.42)
Primary/Lower secondary	1.03 (0.65–1.64)	0.85 (0.50–1.43)	0.63 (0.35–1.15)	0.55 (0.29–1.04)	0.69 (0.44–1.07)	0.65 (0.41–1.03)	1.14 (0.64–2.04)	1.04 (0.56–1.94)	1.19 (0.77–1.84)	1.18 (0.74–1.89)
Marital status (Ref. cat. – Married/Partnership)										
Single/divorced/widowed	1.04 (0.78–1.37)	1.07 (0.77–1.48)	0.99 (0.72–1.37)	0.94 (0.65–1.35)	1.08 (0.82–1.41)	1.09 (0.81–1.47)	1.30 (0.91–1.86)	1.05 (0.69–1.59)	1.53 * (1.17–1.99)	1.08 (0.81–1.45)
Affected mental health^d^										
Yes	4.86 ** (3.62–6.53)	4.82 ** (3.53–6.58)	3.28 ** (2.39–4.49)	3.06 ** (2.20–4.24)	1.78 ** (1.31–2.42)	1.67 * (1.21–2.28)	3.24 ** (2.27–4.63)	2.83 ** (1.95–4.11)	2.18 ** (1.65–2.89)	1.95 ** (1.45–2.61)
Increased work demands										
Yes	1.78 ** (1.32–2.41)	1.77 * (1.26–2.47)	1.55 * (1.10–2.17)	1.49 * (1.04–2.13)	1.55 * (1.13–2.14)	1.59 * (1.13–2.21)	1.00 (0.66–1.51)	0.96 (0.62–1.49)	0.73 * (0.54–0.99)	0.74 (0.53–1.02)
Decreased personal income										
Yes	2.06 ** (1.44–2.94)	1.90 * (1.27–2.83)	1.32 (0.88–1.98)	1.19 (0.77–1.84)	1.19 (0.82–1.73)	1.19 (0.80–1.76)	1.81 * (1.17–2.81)	1.52 (0.95–2.42)	1.65 * (1.16–2.34)	1.54 * (1.06–2.24)
Living with someone <age 15										
Yes	1.25 (0.96–1.64)	1.18 (0.86–1.62)	1.24 (0.91–1.67)	1.27 (0.90–1.79)	1.20 (0.92–1.55)	1.25 (0.94–1.67)	1.07 (0.75–1.51)	0.95 (0.63–1.42)	0.61 ** (0.47–0.78)	0.62 * (0.46–0.83)

*p < 0.05, **p < 0.001.

a The addictive behaviours include smoking and alcohol use.

b The sedentary behaviours include time spent watching TV and time spent playing digital games.

c Multivariable (adjusted) regression models analysed for all of the listed variables.

d Affected mental health is defined as 4 (“bad”) or 5 (“very bad”) point on a Likert scale.

#### Dietary Habits

As Table 4 shows, compared to a middle-aged and elderly, the youngest respondents (aged 15–29) were more likely to worsen their dietary habits. Also, people with increased work demands and people with affected mental health were associated with worse dietary habits that were reported during the pandemic. After adjusting, the protective effect of age was only significant for the oldest age group (aged 60+). Similarly, the strongest association was observed in people whose mental health had deteriorated (OR = 3.06; CI: 2.20–4.24).

#### Physical Activity

Consistent with the results of the dietary habits, people with affected mental health and people with increased work demands due to the pandemic were more likely to report less physical activity. The protective effect of age (60+) was no longer significant after adjusting for the other variables (Table 4).

#### Addictive Behaviour

As Table 4 shows, the youngest respondents (aged 15–29) and people with affected mental health were more likely to be affected by the addictive lifestyle factors (smoking and alcohol consumption). By contrast, people aged, 45–59 and 60+ were significantly less associated with unhealthy behaviours than the youngest age group (OR = 0.36; CI: 0.21–0.61; OR = 0.21; CI: 0.10–0.43, respectively).

#### Sedentary Behaviour

Similarly to the previous results, the youngest age group and people with decreased personal income due to the COVID-19 pandemic were more likely to increase time spent by watching TV or playing games, compared to the older age groups. In addition, caring for children aged <15 was associated with lower adherence with sedentary behaviours (OR = 0.62; CI: 0.46–0.83) in the full-adjusted model (Table 4).

## Discussion

In two cross-sectional surveys that were conducted in the Czech Republic in Spring 2020 and Autumn 2020, we investigated self-reported mental health changes during two periods of lockdown. The major findings of this study indicate that the COVID-19 pandemic has had a negative impact on public mental health, which has decreased significantly over time.

These two studies showed an escalating mental health crisis during the pandemic, which is in accordance with the results of a South Korean study [10]. Moreover, the results of our subsequent survey regarding the major risk factors that affected mental health are consistent with our previous findings, which supports the research evidence that showed a negative effect of being female, young, person with increased work demands and decreased personal income [18]. The results are also comparable with the recent Czech cross-sectional study showed that younger adults, students, those having lost a job or on forced leave displayed high prevalence of mental health [38]. Moreover, these results are also comparable with a United Kingdom cross-sectional cohort study that found an association between worsened mental health and being female and a young age [17]. It could, of course, be argued that gender and age are recognised risk factors for mental health [39], but it should be emphasised that almost three quarters (73.6%) of the mental health-affected respondents reported that the main reason for this change was the long-term social isolation, lack of meaningful activities, concern about a relative’s health and the fear of an uncertain future and a decrease in income, which are consequences that are specifically associated with the adopted COVID-19 measures. Therefore, a significant effect of other factors that were not COVID-19 related was not considered. This assumption is supported by the findings of a German longitudinal study of occupational psychology. The study demonstrated the effect of the duration of the pandemic on the mental health of working women that was significantly impacted by exhaustion due to closures of childcare facilities, increased demands at work and job insecurity [40]. Compared to the first observation, the association between decreased personal income and affected mental health strengthen in the second observation. The possible explanation might be related to people’s concerns about the future during the long-lasting pandemic regarding their financial stability. Interestingly, the affected mental health observed in this study was not being likely the result of the high concerns about suffering for COVID-19 as the risk perception decreased significantly in spite of increasing incidence and mortality in the Czech Republic. With respect to the unforeseen change of daily lives due to COVID-19 lockdown, we examined the healthy and unhealthy lifestyle behavioural changes in the second observation. The most significant unhealthy behavioural change was reported by people who indicated an increased frequency of watching TV/movies or playing digital games. A similar COVID-19 consequence has recently been reported in numerous studies from around the world [40]. Our results also showed an increase in the frequency of smoking, while the factors that are associated with the consumption of alcohol did not show any clear tendency. We identified sociodemographic, mental health and COVID-19 determinants that might contribute to lifestyle behavioural changes. Specifically, we found that individuals with affected mental health were more likely to report an increase in the frequency of unhealthy behaviours than individuals whose mental health was not impacted. We also found that an increase in the frequency of addictive and sedentary behaviour was associated with a younger age and decreased personal income. Together, these findings provide important evidence of the specific groups of people who might be at substantial risk for adopting addictive and unhealthy behaviours. These results are comparable with the recent studies that showed that people with poor mental health were more likely to report increased alcohol consumption [26]. Additionally, the increased time spent on social media or playing digital games was also associated with affected mental health, especially in adolescents and young adults in several studies [41–43]. At this point, it should be emphasised that the cross-sectional nature of the data impeded to investigate a direction of causality. Therefore, we might consider that worsened mental health could contribute to an increase in the frequency of unhealthy behaviours or could be a possible consequence of the over-consumption/over-use of these types of behaviours.

Consistent with the previously published COVID-19 related research, we detected a substantially worsened healthy lifestyle behaviours such as quality of sleeping, dietary habits and physical activity. Specifically, two thirds of the respondents reported decreased frequency of their physical activity, one in five people reported worsened dietary habits and nearly one in three people suffered from a lack of quality of sleeping. These findings are comparable with other current studies. For example, the COVID-19 lockdown has been linked to poor sleep quality in United Kingdom, China and Italy [25, 34–36], a negative change was reported for physical activity in an Australian adult study [37] and people reported more snacking and an increase of their food intake in a recent systematic review [30].

All of these unhealthy lifestyle changes are particularly associated with individuals with affected mental health and increased work demands. These findings are consistent with numerous studies that have examined the association between healthy lifestyle behaviours and psychological and mental factors. For example, a significant inverse impact of a low level of physical activity on depression and anxiety was reported in a meta-analysis of 92 studies that was published before the COVID-19 pandemic [37]. In addition, people with a reduced personal income were more likely to report a worsened quality of sleep and the youngest group of respondents was significantly affected by poor dietary habits compared to the elderly.

### Strengths and Limitations

This study had several limitations. The cross-sectional nature of data did not permit the causality of the examined associations to be determined. Therefore, there are number of potential inverse relationships in our study as was mentioned above. Moreover, our surveys in Spring 2020 and Autumn 2020 were not taken by the same group of people but from independent samples that led to various proportion of respondents within the gender and age groups. Specifically, the second wave of the survey provided sample with lower proportion of women (66.5 vs. 76.8%) and young people aged 15–29 years (15.5 vs. 25.2%) compared to the first observation. Due to these demographic differences among Spring and Autumn samples, the results might be possibly biased. Next, we collected data *via* an online-based questionnaire that excluded the possibility of verifying the data and may limit specific social groups from the study due to internet connection necessity. Moreover, no quotas for data stratification by age, gender or education were used, thus the representative sample was not achieved. The respondents of this study attained higher educational level and were more frequently married than average Czech population. In addition, no standardized questionnaire that has been generally utilized to assess mental health, was used. Therefore, it might be assumed that the comparability of the obtained results will be aggravated. Another limitation is associated with the fact that we did not evaluate specific mental outcomes (e.g., anxiety, depression or loneliness), and therefore, the demonstrated results are generalised and should be verified for specific psychological effects in the future. Furthermore, no positive effects of the pandemic on healthy lifestyle changes were analysed; however, several studies have reported interesting positive changes in the dietary habits related with a more frequent consumption of comfort food, fruits and vegetables [30].

This study also has some notable strengths. To the best of our knowledge, this is one of the first studies that provides a time-related investigation of the changes in mental health and identifies its sociodemographic, economic and occupational determinants. It is also noteworthy that the timing of data collection was accurately set up in respect to the adoption of the lockdown restrictions in the Czech Republic. Moreover, we collected a large sample size and included multiple healthy and unhealthy behaviours for our analyses. Finally, the socioeconomic consequences of the COVID-19-related lifestyle and mental health changes that were demonstrated in this presented study will provide an important source of information that will be valuable in the current cohort study of Healthy Aging in Industrial Environment (HAIE) project in order to understand the long-term health implications in the Czech population [44].

### Conclusion

Our results suggest that young people, those with increased work demands and decreased personal income might be at a high risk of being affected, firstly, by worsened mental health; secondly, by adopting unhealthy lifestyle behaviours and lastly, by the resulting non-communicable diseases. Our results also suggest that there was probably a lack of effective recommendations that emphasised the importance of maintaining physical activity, healthy dietary habits and high quality of sleeping for physical and mental health in the Czech population. Therefore, the health promotion strategies that are directed to people at risk should be encourage individuals to adopt or maintain positive health-related behaviours.
