# Supplementary material for: Self-Reported Mental Health and Lifestyle Behaviour During the COVID-19 Pandemic in the Czech Population: Evidence From Two Cross-Sectional Surveys
Source: Int J Public Health. 2022 May 12;67:1604395. doi: 10.3389/ijph.2022.1604395 (PMC9134237; doi:10.3389/ijph.2022.1604395)
Supplement: Supplementary file 1 [file DataSheet1.docx]

SUPPLEMENTAL MATERIAL 1

Phrasing of the question related to reason of the affected mental health:

“What is the reason of your worsened mental health?”

- Reduce of work and leisure activities - lack of meaningful activity
- Change of household routines due to significantly more time spent together
- Adaptation to changes in daily routines and work habits in the context of home-office
- Overload of work responsibilities combined with household/children's education
- Impaired relationships with other family members during emergency measures
- Isolation from family and friends, reduced social ties
- Fear of limited/unavailable health care
- Concerns about close person’s health
- Sudden death of a close person
- Concern about job loss
- Concern about reduction in personal or family income
- Concern about reduction in value of savings
- Concerns about uncertain future
- Other: (*Please, describe)*

SUPPLEMENTAL MATERIAL 2

Individual and Socio-economic variables characteristics:

Ordinal logistic regression models were conducted to analyse whether the individual demographic and socio-economic factors (gender, age, education, marital status, increased work demands, decreased personal income and living with a child < aged 15) were associated with mental health. All these variables together with the variable “affected mental health” were used to examine the association with behavioural lifestyle changes.

The variables were assessed as categorical variables. Age was first collected as a continuous variable that was cut-off in 15 years of age and consequently categorized into four separate groups. The education contained five separate categories (primary, lower secondary, upper secondary, tertiary, university) that were finally grouped into three the most frequent educational categories (primary/lower secondary; upper secondary; university/tertiary). Also marital status specified five separate categories (single, married, in partnership, divorced, widowed) that were consequently grouped into two categories characterized whether the respondent live alone or not (married/partnership; single/divorced, widowed).

Reference categories were chosen based on commonly used approach. In case of ordinal variables, the lowest one was selected as reference (e.g. lowest age, lowest educational category etc.). In case of categorical variables, less risky category was chosen as reference (e.g. being married/in partnership is considered to be in lower risk of mental health issues).
